# Studies on the changes of uPA system in a co-culture model of bone marrow stromal cells–leukemia cells

**DOI:** 10.1042/BSR20194044

**Published:** 2020-11-19

**Authors:** Lanxia Zhou, Hong Guo, Fang Jia, Xuan Chen, Xiaowei Zhang, Shouliang Dong, Li Zhao

**Affiliations:** 1 The Central Laboratory, The First Hospital, Lanzhou University, Lanzhou, Gansu, China; 2 Critical Care Medicine Department, The First Hospital, Lanzhou University, Lanzhou, Gansu, China; 3 Institute of Biochemistry and Molecular Biology, School of Life Sciences, Lanzhou University, Lanzhou, Gansu, China; 4 Key Laboratory of Preclinical Study for New Drugs of Gansu Province, Lanzhou University, Lanzhou, Gansu, China

**Keywords:** HL60, HS-5, K562, matrix metalloproteins-9, vascular endothelial growth factor

## Abstract

The core of the tumor microenvironment in the hematological system is formed by bone marrow stromal cells (BMSCs). In the present study, we explored the interaction between the urokinase plasminogen activator (uPA) system and the leukemia bone marrow microenvironment (BMM). We established BMSCs–HL60 and HS-5–K562 co-culture models in direct contact mode to simulate the BMM in leukemia. In BMSCs-HL60 co-culture model, the expression levels of uPA, uPA receptor (uPAR), plasminogen activator inhibitor 1 (PAI-1) and vascular endothelial growth factor (VEGF) in BMSCs were higher than those in mono-cultured BMSCs. Matrix metalloproteinase (MMP)-9 (MMP-9) was up-regulated in co-cultured HL60 cells. In HS-5–K562 co-culture model, only uPA, PAI-1, and VEGF-A were up-regulated in HS-5 cells. The levels of the uPA protein in the co-culture supernatant were significantly higher than that of mono-cultured BMSCs or HS-5 cells. Our findings demonstrate that the co-culture stimulates the production of uPA, uPAR, PAI-1, MMP-9, and VEGF-A by BMSCs. It could further explain how the uPA system in leukemia cells is involved in the growth, development, and prognosis of leukemia.

## Introduction

The urokinase plasminogen activator (uPA) system consists of uPA, its cognate receptor (uPAR) and two specific inhibitors, the plasminogen activator inhibitor 1 (PAI-1) and 2 (PAI-2) [1]. This system plays a key role in the degradation of the extracellular matrix (ECM) and basement membrane, stimulating tumor cell invasion and metastasis and allowing malignant cells to invade locally and eventually spread to distant sites [2]. uPA is synthesized as pro-uPA, a single chain consisting of three domains (growth factor-like domain, kringle domain, and serine protease domain) [3]. The almost inactive pro-uPA could be cleaved and activated into a two-chain form by plasmin, cathepsin B, kallikrein, or other hydrolyzing factors [4]. uPAR, which consists of D1, D2 and D3 domains, is anchored to the cell surface by glycosylphosphatidylinositol-anchored protein and has a high affinity for uPA and pro-uPA [5]. Due to the lack of transmembrane and intracellular domains, uPAR must bind to co-receptors such as integrins, G-protein-coupled receptors, and growth factor receptors to activate cell motility, proliferation, and survival [6,7]. PAI-1 and PAI-2 are two inhibitors of the uPA system, which belong to the serine protease inhibitor family. It is now apparent that uPA and PAI-1 interact with several cell surface receptors, transducing intracellular signals that significantly affect both motility and proliferation. The active form of uPA transforms plasminogen into plasmin, which cleaves and activates matrix metalloproteinases (MMPs). The activated MMPs cause ECM degradation and the release of various growth factors to promote the migration of tumor cells [2]. Vascular endothelial growth factor (VEGF) promotes angiogenesis by stimulating the migration of individually cultured endothelial cells, inducing the phosphorylation and redistribution of the adherent linker molecules, or increasing the proteolytic enzymes [8]. Studies have shown that VEGF could promote the expression of uPAR in vascular endothelial cells [9]. A recombinant KDRscFv-uPAcs-Lβ-KDEL which has therapeutic applications on uPA-overexpressing tumors was established [10].

The tumor microenvironment of the hematological system is the bone marrow microenvironment (BMM), which includes the bone marrow stromal cells (BMSCs), the ECM components and the soluble factors secreted by the stromal cells. The core is formed by the BMSCs [11]. The BMM serves as the site of leukemia initiation and progression and is also the most common relapse site of leukemia [12]. There is growing evidence that the occurrence of leukemia and the response to chemotherapy are severely affected by the interaction with the BMM [13]. The microenvironment has a pro-active role in the regulation of the signaling enhancer and pro-survival molecule lymphoid proto-oncogene *TCL1* in chronic lymphocytic leukemia [14]. Studies have shown that high expression levels of uPAR can be observed in acute myeloid leukemia (AML) and that this is associated with poor prognosis [15]. Elevated levels of uPA and PAI-1 were detected in patients with chronic myeloid leukemia. Therefore, it is necessary to study the role of the uPA system in the leukemia BMM and to investigate its impact on clinical outcomes.

Dr. Sipkins summarized the role of the BMM in leukemia progression and treatment, posing more unanswered questions [16]. There is an extension of 2D co-culture established wherein acute lymphoblastic leukemia (ALL) cells uniquely interact with BMSCs. The ‘phase dim’ ALL exhibits a unique phenotype and shows more efficiency in pre-clinical design and investigation [17]. In this study, the BMSCs obtained from leukemia patients were used to simulate the microenvironment of leukemia. Using direct-contact culture methods [18], we established the co-culture model of BMSCs–leukemia cells. Moreover, we explored the expression of uPA, uPAR, PAI-1, MMP-9, and VEGF-A under co-culture conditions and the effect of the uPA system on the BMM during leukemia. The present study aims to provide a basic understanding of the uPA system and to clarify the relationship of the BMM and the recurrence and poor prognosis of leukemia.

## Materials and methods

### Cell culture

The leukemia cell lines K562 and HL60 (K562 and HL60 were from the stock of the Central Laboratory of First Hospital, Lanzhou University) [19] were grown in RPMI-1640 medium (HyClone) supplemented with 10% fetal bovine serum (FBS, PAN-Biotech, Germany) and 1% antibiotics (penicillin, streptomycin). The HS-5 cell line (JENNIO Biological Technology, China) was grown in DMEM/F12 medium (GIBCO) supplemented with 15% FBS and 1% antibiotics (penicillin, streptomycin). The BMSCs were obtained from B-cell acute lymphoblastic leukemia (B-ALL) patients (age, 6–64 years; median age, 17.8 years; eight males and two females), who were enrolled from Central Laboratory of First Hospital, Lanzhou University (Lanzhou, China). All conditions were confirmed by pathological examination and all patients signed informed consent. The experiments met the ethical standards of human experimentation and were approved by the ethics committee of The First Hospital of Lanzhou University (LDYYLL2017-98). Human BMSCs (HBMSCs) were isolated and cultured according to the method described previously [20]. Briefly, under sterile conditions, 2 ml fresh bone marrow blood was extracted from the B-ALL patients. Subsequently, the bone marrow was added to 4 ml Lymphocyte Separation Medium (MP Biomedicals, America) separation at 2100 rpm for 20 min at room temperature. The single nucleus layer was transferred to another centrifuge tube, and washed twice with 10 ml phosphate-buffered saline (PBS). The cells were seeded in 10 ml DMEM/F12 (GIBCO) medium (containing 15% FBS and 1% antibiotics penicillin–streptomycin). The cells were cultured at a density of 1 × 10^5^ /ml in 5% CO_2_ incubator (Thermo, America) at 37 °C. The medium was changed after 5 days and the non-adherent cells were removed gently. Subsequently, the medium was changed every 2–3 days. The adherent cells were expanded until 80–90% confluence was reached.

### Co-culture

HS-5 cells/the fourth passages (P4) HBMSCs were digested for 2 min with 0.25% Trypsin/EDTA (GIBCO), 10 ml PBS was used to terminate the digestion, the solution was centrifuged at 1000 rpm for 5 min, and the supernatant was removed. The cells were seeded in the 25-cm^2^ cell culture flask at a concentration of 5 × 10^4^ cells/ml in 9 ml DMEM/F12 plus 10% FBS. After 24 h, the cells were totally adherent. Then 1 ml (1 × 10^4^ cells/ml) of K562 were cultured in direct contact with HS-5 cells; 1 ml (1 × 10^4^ cells/ml) HL60 cells were cultured in direct contact with P4 HBMSCs in culture flask. The total co-culture system was 10 ml. Monocultures of BMSCs, HS-5, HL60 and K562 cells were seeded at the same above-mentioned conditions as controls. K562 cells were harvested after incubation of 3 and 4 days, HL60 cells were harvested after incubation of 3 and 5 days. The supernatant was used for enzyme-linked immunosorbent assay (ELISA) test. The precipitated K562 and HL60 cells in the culture system after centrifugation were used to extract RNA. The digested BMSCs and HS-5 in co-culture were used to extract RNA.

### Identification of HBMSCs

Human CD34-phycoerythrin (PE, BD 652802), CD44-fluorescein isothiocyanate (FITC, LOT E00298-1631 eBioscience) monoclonal antibodies and their isotype controls in BMSCs of co-culture system were analyzed by Flow cytometry as previously described [20].

### Quantitative real time polymerase chain reaction

The total RNA was prepared from each cells using RNAiso Plus Reagent (Takara, Japan) according to the manufacturer’s guidelines. Complementary DNA (cDNA) was synthesized and data were analyzed as previously reported [21]. Primers of uPA, uPAR, PAI-1, VEGF-A and MMP-9 (all from Takara, Japan) were used, the sequences are in Table 1. The levels of uPA, uPAR, PAI-1, VEGF and MMP-9 were normalized against those of β-actin mRNA.

**Table 1:** Real-time quantitative PCR primers

Genes		Sequence (5′→3′)
*β-actin*	F	TGGCACCCAGCACAATGAA
	R	CTAAGTCATAGTCCGCCTAGAAGCA
*uPA*	F	CTCCAACATTCACTGGTGCAACT
	R	CATCAGATCTGTGGGCATGGT
*uPAR*	F	AACAGTGCCTGGATGTGGTG
	R	GAAGTGGAAGGTGTCGTTGTTG
*PAI-1*	F	AATGACTGGGTGAAGACACACACA
	R	TTCCACTGGCCGTTGAAGTAGA
*VEGF-A*	F	GAGCCTTGCCTTGCTGCTCTA
	R	CACCAGGGTCTCGATTGGATG
*MMP-9*	F	GCTACGTGACCTATGACATCCTG
	R	CCTCCACTCCTCCCTTTCCT

### ELISA

The level of secreted uPA was quantified using the uPA ELISA Kit. Briefly, cell supernatant from 3 and 4 days mono-cultured or co-cultured cells were detected at 450 nm by a Microplate Reader (Thermo, America).

### Statistical analysis

Microsoft Excel and GraphPad Prism 5 software were used for the statistical tests. All data were recorded as means ± SEM. Difference was considered significant when *P*<0.05. The homogeneous variances among groups were analyzed by one-way analysis of variance (ANOVA) followed by the Bonferroni’s post hoc test.

## Results

### Development of co-culture system for leukemia cells and BMSCs

**Figure 1:**
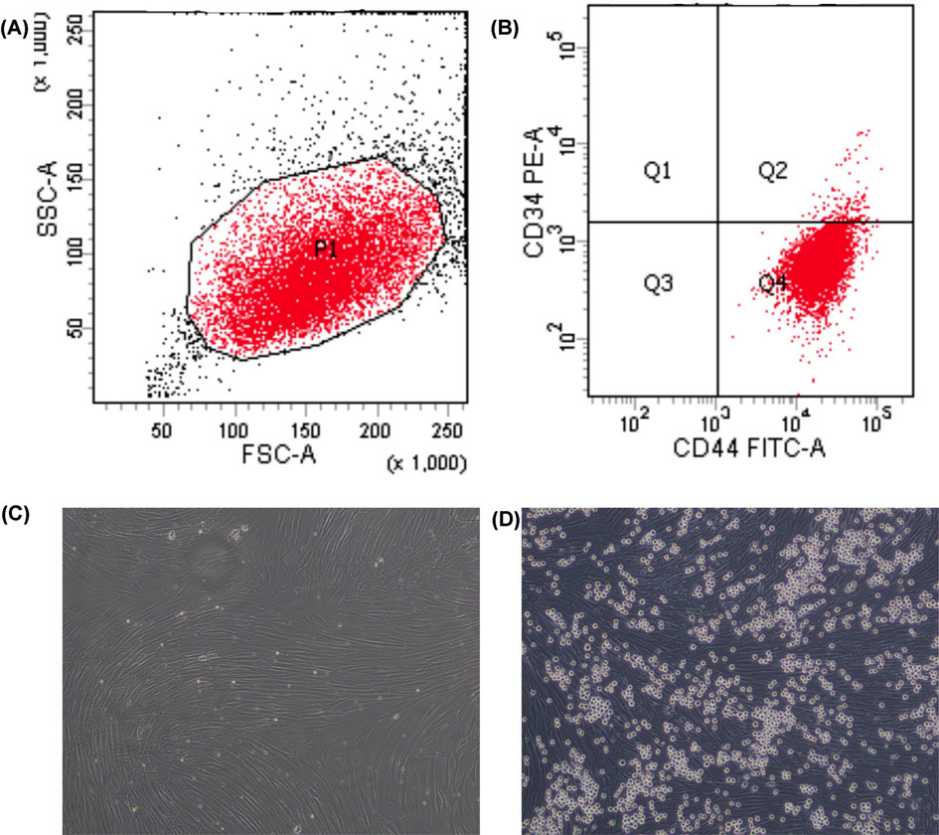
Flow cytometry analysis of cell surface markers of BMSCs and light microscopy observation of BMSCs and a BMSC–HL60 co-culture (**A**) P1, the gated target cells. (**B**) Q4, positive marker CD44 and negative marker CD34 of BMSCs. (**C**) BMSCs from B-ALL patients demonstrated a spindle-shaped morphology. (**D**) Co-cultures of BMSCs and HL60 cells after 3 days (original magnification, ×100).

We used Forward Scatter and Side Scatter to gate the target cells. When the target cells were sorted by flow cytometry, it revealed that these BMSCs expressed positive surface marker CD44, and the negative surface marker CD34. These results confirmed that the selected cells were BMSCs (Figure 1A,B). BMSCs were collected from patients diagnosed with B-ALL. BMSCs derived from the B-ALL patients, BMSC–HL60 co-cultures, and HS5–K562 co-cultures were grown in the same culture medium and could be split several times. BMSCs were observed in the logarithmic growth phase under an inverted microscope. Cells growing in adherent, fibrous, swirling, or radial arrangements transformed to present a spindle-shaped morphology. The cells showed a high degree of homogeneity (Figure 1C). BMSCs co-cultured with HL60 cells were observed under the inverted microscope, we saw that the HL60 cells adhered to BMSCs and grew well. The BMSCs were spindle-shaped and had become slender (Figure 1D).

### Expression of uPA, uPAR, PAI-1, MMP-9, and VEGF mRNA in BMSC–HL60 co-culture

**Figure 2:**
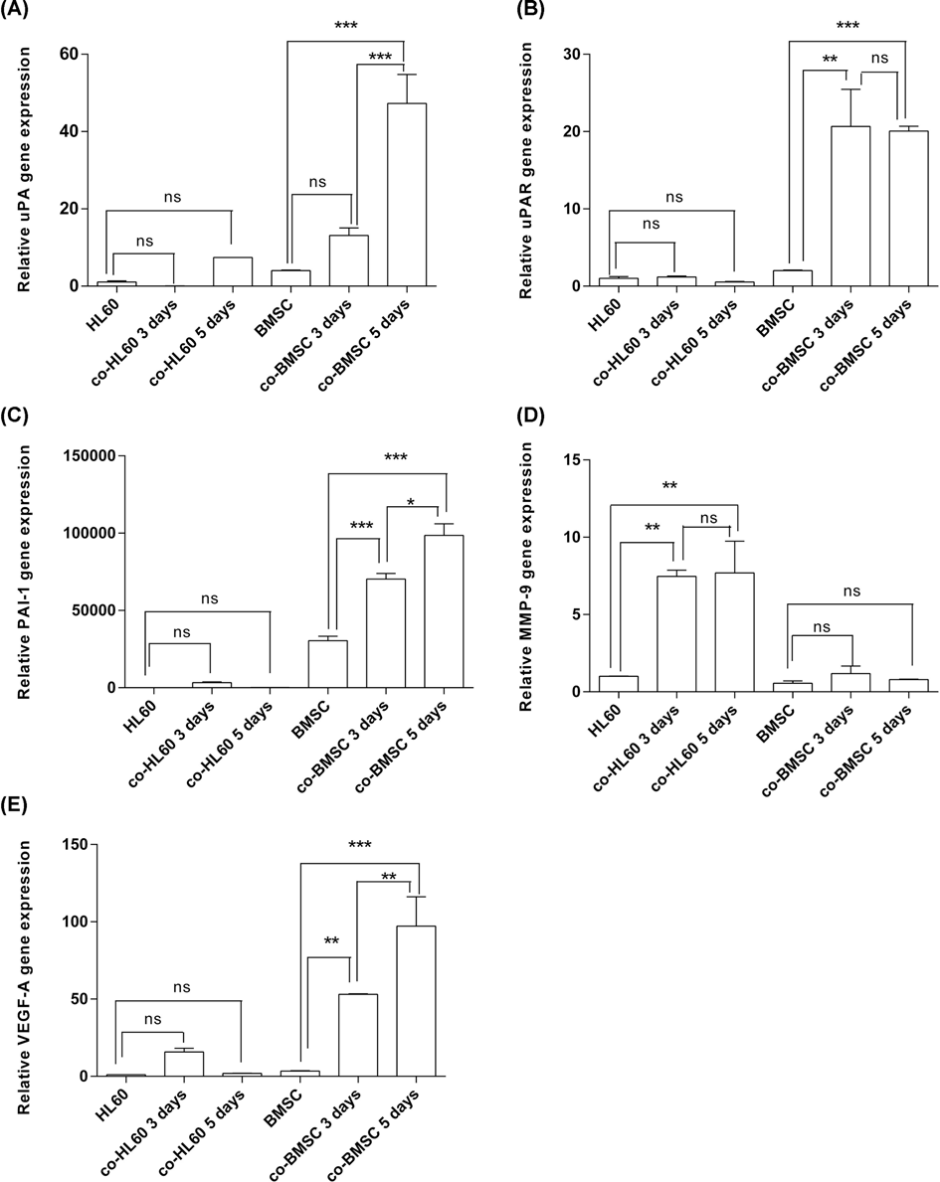
Relative mRNA expression levels of uPA, uPAR, PAI-1, MMP-9, and VEGF-A in BMSCs, HL60 cells, co-cultured BMSCs (with HL60s) and co-cultured HL60 cells (with BMSCs) after being cultured for 3 and 5 days (**A**) uPA, *n*=2. (**B**) uPAR, *n*=2. (**C**) PAI-1, *n*=2. (**D**) MMP-9, *n*=2. (**E**) VEGF-A, *n*=2. The bars represent mean ± SEM. ***P<0.001; **P<0.01; *P<0.05; ns, not significant.

To assess whether the mRNA expression levels of uPA, uPAR, PAI-1, MMP-9, and VEGF-A were up-regulated in other leukemia cells co-cultured with BMSCs, we co-cultured BMSCs with HL60 and checked the expression of uPA, uPAR, PAI-1, MMP-9, and VEGF in the cells by quantitative real time polymerase chain reaction (Q-PCR). The results are shown in Figure 2. We observed that the expression levels of uPA, uPAR, PAI-1, and VEGF in co-cultured BMSCs were significantly higher than those in monocultured BMSCs (Figure 2A–C,E). We also found that the expression levels of uPA, PAI-1, and VEGF-A were significantly higher in 5 days’ co-cultured BMSCs than that of 3 days. In contrast, MMP-9 was expressed in co-cultured HL60 cells but not in co-cultured K562 cells (Figure 2D).

### Expression levels of uPA, uPAR, PAI-1, MMP-9, and VEGF mRNA in HS-5–K562 co-culture

**Figure 3:**
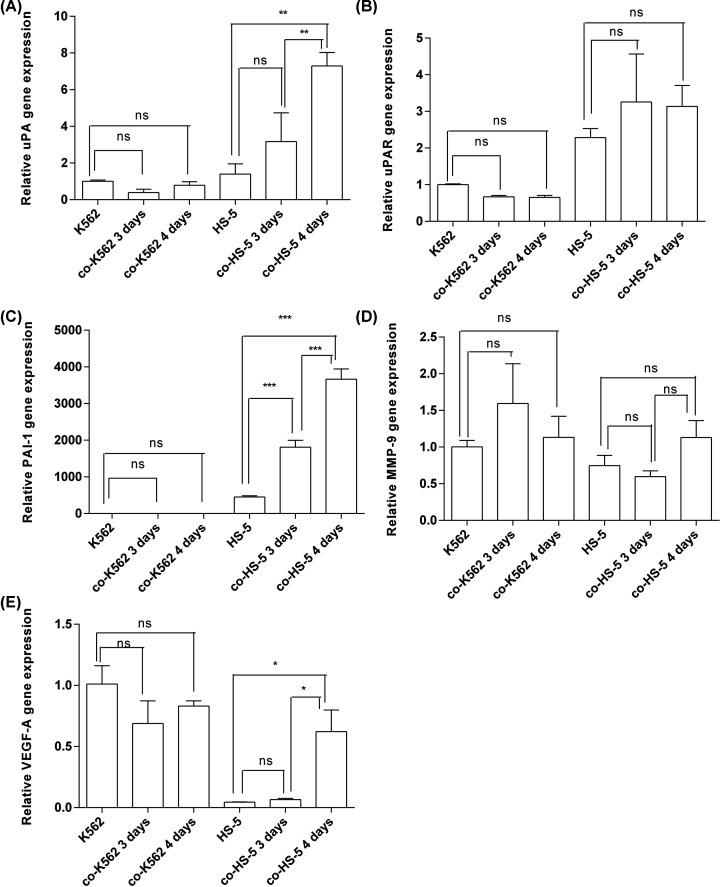
Relative mRNA expression levels of uPA, uPAR, PAI-1, MMP-9, and VEGF-A in HS-5 cells, K562 cells, co-cultured HS-5 (with K562) and co-cultured K562 (with HS-5) after being cultured for 3 and 4 days (**A**) uPA, *n*=2. (**B**) uPAR, *n*=2. (**C**) PAI-1, *n*=2. (**D**) MMP-9, *n*=2. (**E**) VEGF-A, *n*=2. The bars represent mean ± SEM. ***P<0.001; **P<0.01; *P<0.05; ns, not significant.

Our results show that the up-regulated expression of uPA, uPAR, PAI-1, and VEGF-A were observed in co-cultured BMSCs, which was obtained from leukemia patient (Figure 2). Furthermore, the BMSC line HS-5 was co-cultured with K562 cells to explore the interaction of leukemia cells and healthy HBMSCs. As shown in Figure 3, the mRNA expression levels of uPA, PAI-1, and VEGF-A were higher in co-cultured HS-5 (Figure 3A,C,E); while uPAR and MMP-9 expression levels did not reveal any significant changes in either cell line (Figure 3B,D).

### Protein expression of uPA in co-culture

**Figure 4:**
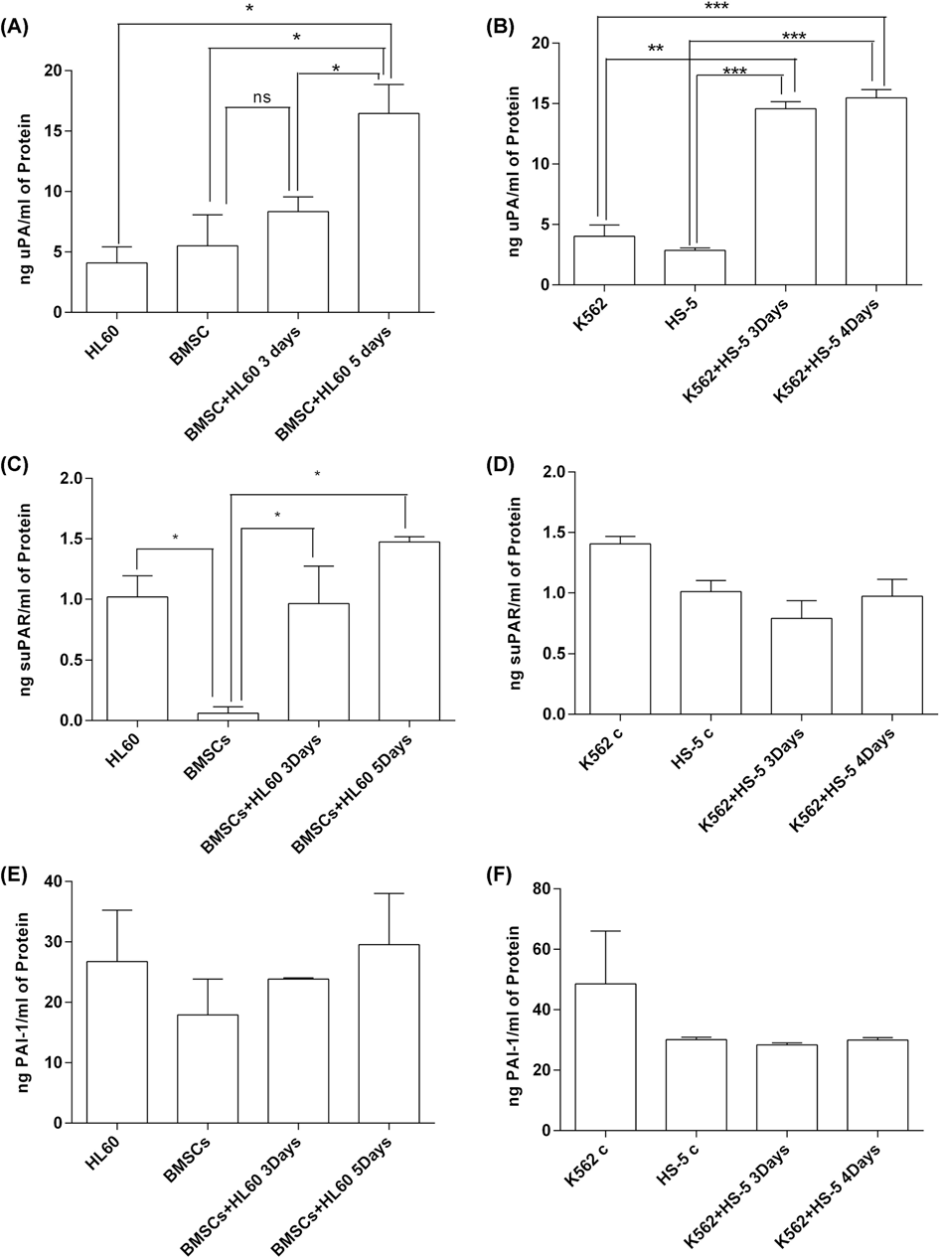
Protein expression levels of uPA, uPAR, and PAI-1 in BMSCs-HL60 co-culture and K562-HS-5 co-culture (**A**) uPA in BMSCs-HL60, *n*=2. (**B**) uPA in K562-HS-5 co-culture, *n*=2. (**C**) uPAR in BMSCs-HL60, *n*=2. (**D**) uPAR in K562-HS-5 co-culture, *n*=2. (**E**) PAI-1 in BMSCs-HL60, *n*=2. (**F**) PAI-1 in K562-HS-5 co-culture, *n*=2. The bars represent mean ± SEM. ***P<0.001; **P<0.01; *P<0.05; ns, not significant.

To explore the protein expression levels of uPA system in the supernatants from BMSCs and HS-5 cells co-cultured with leukemia cells, we measured the protein levels of uPA, suPAR, and PAI using ELISA. We harvested the supernatants from the co-cultures and monocultures at different time points. Our results show that the uPA protein level in co-culture supernatants were significantly higher than that of BMSCs or HS-5 or K562 or HL60 monocultures (Figure 4A,B). Moreover, suPAR protein level in BMSCs-HL60 co-culture instead of K562-HS-5 co-culture was up-regulated notably (Figure 4C,D). While PAI-1 protein level in co-cultures did not have significant changes compared with the monocultures (Figure 4E,F).

## Discussion

The BMM is changed in leukemia and plays critical roles in the leukemia pathogenesis. It is reported that the uPAR expression was elevated in AML patients than healthy people, that primary AML patients with high expression levels of uPAR often receive a poor prognosis and that uPA can be used as an important prognostic indicator for patients with AML [22]. In human cancer, high expression levels of uPA and PAI-1 are also associated with poor prognosis. BMSCs and leukemia cells both contribute to the creation of a competitive niche more favorable for leukemia stem cells [23]. In this leukemia study using BMSC–leukemia cell direct-contact cultures for different time intervals, the gene expression levels of uPA, uPAR, PAI-1, and VEGF-A in co-cultured BMSCs were significantly higher than in monocultures (Figure 2A–C,E). Interestingly, when we co-cultured HS-5 with K562 cells, we found that only uPA, PAI-1, and VEGF-A were up-regulated and that the differences in uPAR expression levels were not significant in both cell lines (Figure 3). Comparing with normal HBMSCs, leukemia cells were demonstrated to more strongly stimulate the expression of uPA, uPAR, PAI-1, and VEGF-A in leukemia BMSCs under co-culture conditions. We further used ELISA to detect the uPA protein levels in the supernatants from BMSCs and HS-5 cells co-cultured with leukemia cells. Compared with the monocultured BMSCs, the uPA protein levels in the supernatant were higher than under co-culture conditions, which is consistent with the observed increase in gene expression of uPA. Li et al. found the expression of uPA and MMP-2 increased in a human umbilical vein endothelial cell (HUVEC)–HBMSC co-culture system, but PAI-1 expression had not significantly increased [21]. This result is consistent with the findings of our study, suggesting that co-culture conditions can promote the expression of these cytokines. By examining IL-17 signaling-related genes, IL-8, CCL2 levels, mTOR signaling and EIF2 signaling pathways genes, it is confirmed that BMSCs and leukemia cells both contribute to the creation of a competitive niche more favorable for leukemia stem cells [23].

Studies have shown that VEGF can promote the expression of uPAR in vascular endothelial cells [9]. In this study we also explored the expression of VEGF-A under co-culture conditions. The results show that the expression of VEGF-A in co-cultured BMSCs and co-cultured HS-5 cells was significantly increased. Li et al. added VEGF neutralizing antibodies to the co-culture system and found that this significantly inhibited the expression of uPA and uPAR in co-cultured HUVECs and co-cultured HBMSCs, indicating that the elevated expression of VEGF_165_ in co-cultured HBMSCs stimulates the expression of uPA in co-cultured HUVECs to promote the formation of self-assembled networks [24]. This may indicate that the expression of VEGF in BMSCs stimulates the expression of the components of the uPA system, but further experiments are required to confirm these results.

In the present study, we investigated the expression of MMP-9 under co-culture conditions. The expression levels of MMP-9 in co-cultured HL60 cells were significantly higher than those in mono-cultured HL60 cells (Figure 2D). The results show that BMSCs could promote the expression of MMP-9 in HL60 cells. MMPs are known to be activated by plasmins, including MMP-3, MMP-9, MMP-12, and MMP-13. These MMPs not only contribute to the remodeling of the ECM, but also to the release of ECM-related growth factors, such as fibroblast growth factor-2, VEGF, hepatocyte growth factor, insulin-like growth factor, epidermal growth factor, and transforming growth factor-β [25]. The release or activation of these growth factors enhances the expression of uPA, uPAR and PAI-1 components by paracrine, as well as other key processes in the development of cancer, such as angiogenesis and epithelial–mesenchymal transition [2,26]. Our results show that BMSCs could promote the expression of MMP-9 in HL60 cells, which may explain the fact that the BMM can contribute to the proliferation of acute leukemia cells. Leukemic cells and cells of the BMM have dynamic interactions in hematological malignancies. The rationale for appropriately tailored molecular therapies targeting not only leukemic cells but also their microenvironment will comes from the complex interplay and ensures improved outcomes in leukemia [27].

## Conclusion

Co-cultures of BMSCs and leukemia cells could time-dependently stimulate the secretion of uPA, uPAR, PAI-1, MMP-9, and VEGF-A, indicating that uPA system plays an important role in the BMM. Because of the complexity of the intracellular signaling pathways, the exact mechanisms by which this secretion is promoted needs to be further studied.

## Data Availability

All the raw data can be obtained from the corresponding author.

## Abbreviations

ALL: acute lymphoblastic leukemia
AML: acute myeloid leukemia
BMM: bone marrow microenvironment
BMSC: bone marrow stromal cell
B-ALL: B-cell acute lymphoblastic leukemia
CCL2: C-C motif chemokine ligand 2
ECM: extracellular matrix
EIF2: eukaryotic initiation factor 2
ELISA: enzyme-linked immunosorbent assay
FBS: fetal bovine serum
HBMSC: human BMSC
HUVEC: human umbilical vein endothelial cell
KDEL: Lys-Asp-Glu-Leu motif
KDRscFv: single chain variable fragment antibody against kinase insert domain‐containing receptor
IL-8: interleukin-8
IL-17: interleukin-17
MMP: matrix metalloproteinase
mTOR: mammalian target of rapamycin
PAI-1: plasminogen activator inhibitor 1
PBS: phosphate-buffered saline
P4: fourth passage
suPAR: urokinase plasminogen activator receptor in supernatants
uPA: urokinase plasminogen activator
uPAR: uPA receptor
VEGF: vascular endothelial growth factor
